# Praxis and autism: the psychomotor regulation sensory processing dimension—a report from the field

**DOI:** 10.3389/fnint.2012.00129

**Published:** 2013-01-18

**Authors:** Kathleen A. Berger

**Affiliations:** ^1^Therapy Intensive Programs, Inc.Tallahassee, FL, USA; ^2^Rehabilitation Science Doctoral Program, College of Public Health and Health Professions, University of FloridaGainesville, FL, USA

A quiet revolution is afoot in our understanding of autism spectrum disorder (ASD). Classically understood as a social disorder (Kanner, 1943) that presents clinically with social and communication difficulty and restricted patterns of behaviors (Lord et al., 2000), both diagnosis and therapeutic interventions have correspondingly focused upon behavioral and typical development theory (Lovaas, 1987; Dawson et al., 2010). Yet recent studies across multiple fields have begun to substantiate what my colleagues and I have come to learn about ASD through almost two decades of clinical work with children. In fact two recent papers propose a cognitive motor model of autism and summarize much of this research (Rizzolatti and Fabbri-Destro, 2010; Mostofsky and Ewen, 2011). That research is further bolstered by (auto) biographical work of several people living with ASD (Williams, 1994; Biklen, 2005; Iversen, 2007). The primary claim is as simple as it is radical ASD has as a primary, defining feature psychomotor regulation sensory processing disorder. Whether this psychomotor dimension simply parallels the social and communication deficits that consume almost all of the attention and resources in research and intervention, or plays an important role in producing those symptoms will have to be the topic of future research over the coming decade. What we can say at present is that an important psychomotor dimension that has etiological and symptomatic aspects exists, and that this has important, if only nascently understood, therapeutic implications.

## Clinical experiences

My professional training as a physical therapist leads me to view things through a physical lens, of sorts. The skeptic is thus justified to wonder about a physical bias. Indeed, my ex-husband, with whom I would frequently discuss my ideas over the past 20 years, served usefully as precisely that critic. I was first introduced to a child with ASD when my then 6-months-old son was diagnosed. After he died at the age of five, I began to work with children through Therapy Intensive Programs, Inc. (TIP)^1^. Between 1995 and 2012, I have partaken in roughly 72 five day sessions working with children ages two through 15. In the past couple of years, we have extended our client base to include young adults (16–20) and most recently adults (21+). I briefly describe the trajectory of my observations and resulting conjectures, none of which would have been possible without the stimulation and support of dozens of TIP team members^2^, as well as the kiddos and their families with whom I have had the pleasure to work.

Initially, therapy at TIP incorporated early work examining movement differences in autism (Donnellan and Leary, 1995), and my observations with my son. Following Donnellan and Leary's work, TIP defines psychomotor regulation disorder as difficulty with initiating, inhibiting, or sustaining a movement, thought or emotion (Damasio and Maurer, 1978; Donnellan and Leary, 1995). Indeed, much animal and imaging research focusing on the repetitive behaviors observed in persons with autism has centered on the frontostriatal pathways. Science currently understands the functional role of frontostriatal pathways is to (1) inhibit a prior thought, action, or emotion (2) select the desired thought, action, or emotion, and (3) inhibit competing thoughts, actions, or emotions. These pathways are differentially connected in persons with autism (Lewis and Kim, 2009).

Further, many recent studies have evidenced asymmetry of connectivity or processing of sensory information in persons with autism, including though not limited to (1) more strongly connected proprioceptive pathways, (2) differential processing of visual information, and (3) attention to multimodal inputs. Paralleling the work by Donnellan and Leary, and more recent neuroimaging and experimental studies, TIP has used “bottom up” sensory pathways to compensate for “top down” cognitive motor difficulties. Examples of bottom up sensory supports include touch cues, amplified proprioception, rhythm, multimodal sensory inputs, and visual supports. We combine these sensory supports with positive behavioral supports. If a child with autism has psychomotor difficulties, then behavior should be viewed as such rather than described as escape, non-compliant, or attention seeking. At TIP, we have found that with provision of sensory supports, movement difficulties are eased. For example, a child who frequently paces becomes able to sit and participate in a 30-min group.

A recent conversation with a special education teacher illustrates interpreting a behavior negatively, and an alternative explanation, based on approaching autism as a cognitive motor sensory processing disorder. “FG,” a participant in our program was a student in this teacher's class. Discussing cognitive movement differences in persons with autism, a teacher described a behavior that she considered intentional. When presented with two choices, FG used both hands and forcefully slapped both choices. Then, after the teacher said “Nice hands,” FG was able to touch one choice.

The teacher had interpreted the behavior as intentional because FG was able to touch the choice following the verbal “reminder.” Alternatively, interpreting FG's behavior as cognitive motor difficulty, reliant on bottom up control, we assume that FG was initially unable to both control his movement and make the choice. Then, after he had made the choice, he was able to control his movement, also facilitated by the “Nice hands” prompt. Alternatively, the teacher could have supported FG by giving him the initial cue to look at the choices, with time to make his decision and then present them again to make his choice. We have found that increased processing time combined with other bottom up sensory supports facilitates controlled movement (Donnellan et al., 2006). Additionally, the language we suggest avoids negative implications. “Ok. FG, get your body ready. My true choice is ….” (present choices).

## Integrating clinical and science

In 1999, I began searching for literature that could offer theoretical and empirical insight to what I was observing in the field. The first article, Teitelbaum et al. (1998) report asymmetrical development of postural and developmental reflexes. In 2006, I returned to graduate school, currently a doctoral candidate in the Rehabilitation Science program at University of Florida. During my tenure in graduate school, several studies have examined sensory and motor learning differences in persons with autism.

## Motor

Recent studies investigated motor learning in high functioning children with autism (HFA) (Cattaneo et al., 2007; Fabbri-Destro et al., 2009; Haswell et al., 2009; Torres, 2012). Cattaneo et al. (2007) investigated oral musculature activity when eating. When a typically developing child first reaches for a cracker, mouth musculature begins to activate. Conversely, this same musculature does not activate until the cracker touches the autistic child's mouth. Other researchers (Fabbri-Destro et al., 2009; Haswell et al., 2009; Torres, 2012) found low spatiotemporal variability in motor learning studies in persons with HFA.

Many children more severely affected with autism have difficulty with fine motor tasks such as eating with a spoon. In chapter 4 of my MS thesis I evaluate home video of a toddler diagnosed with ASD compared to home video of a neurotypical toddler as each eats from a bowl using a spoon^3^. I expected to find highly patterned, repetitive movement tracing the ASD child's hand through space, and random, fluid movement tracing the neurotypical child's hand through space. Though it is only a comparison of two children using judgmental coding techniques of non-standardized home video, the results (p. 35) confirmed this expectation. Additionally, there was little rotation of the forearm for the toddler with autism.

## Sensory processing and accommodations

While these studies evidence movement differences, others evidence differences in sensory processing in persons with autism. For example, recent work by Haswell et al. (2009) indicates that children with autism have “over connectivity” in proprioceptive pathways and are more reliant on these pathways for motor learning. This work parallels our observations at TIP. For example, we observed that our clients respond favorably to resistance or “amplified” proprioception. One easy and simple accommodation we have used at TIP is to provide rhythmic deep squeezes to facilitate maintaining a position. It is a simple recommendation I have made to special education teachers. To illustrate, a teacher expressed concern regarding student safety on an upcoming field trip. The student would frequently run without awareness of safety concerns, and when staff would hold his hand, he would pull. With this one simple accommodation, rhythmic deep pressure, the child was successful and the teacher was happy.

Other studies have suggested that persons with autism preferentially attend to multimodal input. For example, Klin et al. (2009) found that toddlers with autism preferentially attended to audiovisual synchrony over motion cues. This parallels studies evidencing that persons with autism look more at a person's mouth than their eyes (Schultz, 2005). Further (Mizuno et al., 2006), found increased connectivity between the thalamus and the cerebral cortex. Thalamocortical connections are thought to play an important role in intermediating attention (Zikopoulos and Barbas, 2007). At TIP, two examples of where we have observed that multimodal input facilitates attention include (1) audio-tactile input to body parts for motor planning, and (2) synchronizing audio with movement.

For example, we frequently practice different postures in music activities or yoga. Instead of providing a full physical prompt, we combine touch cues and rhythmic auditory cueing, “Go Go Go. You can do it!” For example, if the activity requires that the child get down in a kneeling position, but he is unable, we would tap fast on their knees (tactile) or jiggle their knee facilitating muscle sensory receptors (proprioception) while providing auditory cueing. We have found this support successful in facilitating transition to the desired posture without physically placing the person in the position. Alternatively, an audio cue combined with a visual model has supported independent movement as well.

Though these examples just touch on therapeutic strategies we have found useful at TIP, they illustrate how integrating clinical observation, biographical accounts, and scientific studies can inform and refine treatment methods. Further, recent research agendas urge rehabilitation scientists to look for the active ingredients in therapeutic strategies rather than examining “treatment packages.” Uncovering the active ingredients will optimize dose, frequency, and location of interventions (The American Occupational Therapy Foundation and The American Occupational Therapy Association, 2011; www.aota.org/documentvault/research/45008.aspx). TIP has experienced success using key ingredient sensory support accommodations.

## First person experiences

Though autobiographical accounts of people living with ASD are not scientific evidence, they nonetheless provide useful information we can use to both check, and enhance, new understanding. In particular, two autobiographical accounts illustrate how some individuals experience sensory and motor differences.

[The] knack of knowing where my body is does not come easy for me. Interestingly I do not know if I am sitting or standing. I am not aware of my body unless it is touching something … Your hand on mine lets me know where my hand is. Jarring my legs by walking tells me I am alive.—*Chandima Rajapatirana in Wallis (2006)*.

To think about it, I recall that I learnt every skill through the touch method. I have a problem imitating any movement by looking at people performing or by mapping the instructions given me …. The simple task of holding a spoon and taking food to my mouth was also taught by my speech therapist for by helping me for the first few times till my habit developed …*Tito Rajarshi Mukhopadhyay in Biklen (2005)*.

## Conclusion

It is important to emphasize the extent to which this research is being done by scholars working in different disciplines and publishing in journals with often rather distinct audiences. Put starkly, much of this work is being done in semi-isolation with few of the researchers aware of the full breadth of relevant work being done by others, and here I am only speaking to the work being done by scholars. The current gap between these sundry researchers and the therapists, special education professionals, medical doctors, and others working with the ASD population is a veritable chasm. In the early stages of a revolution both are inevitable. As more of us come to understand ASD as having an important psychomotor dimension this will change, and as that happens an exciting, vibrant field will emerge. I look forward to contributing as a scholar who can bring clinical experience to the field, and hope to also play a role helping to translate the findings in the more technical work so that it is accessible to those working in therapeutic settings.

## Footnotes

^1^TIP is also called Kris' Camp http://www.kriscamp.org/, named in memory of my son.

^2^I especially owe debts to Suzanne Oliver and Michelle Welde Hardy (Neurologic Music Therapy Services of Arizona http://www.nmtsa.org/).

^3^The thesis can be found online at http://kriscamp.org/news/index.html.

## References

[B1] Biklen D. (2005). Tito rajarshi mukhopadhyay and douglas biklen, in Autism and the Myth of the Person Alone, eds Fine M. Marecek J. (New York; London: New York University Press), 116

[B2] Cattaneo L. Fabbri-Destro M. Boria S. Pieraccini C. Monti A. Cossu G. (2007). Impairment of actions chains in autism and its possible role in intention understanding. Proc. Natl. Acad. Sci. U.S.A. 104, 17825–17830 10.1073/pnas.0706273104 17965234 PMC2077067

[B3] Damasio A. R. Maurer R. G. (1978). A neurological model for childhood autism. Arch. Neurol. 35, 777–786 10.1001/archneur.1978.00500360001001 718482

[B4] Dawson G. Rogers S. Munson J. Smith M. Winter J. Greenson J. (2010). Randomized, controlled trial of an intervention for toddlers with autism the early start denver model. Pediatrics 125, e17–e23 10.1542/peds.2009-0958 19948568 PMC4951085

[B5] Donnellan A. M. Leary M. R. (1995). Movement Differences and Diversity in Autism/Mental Retardation Appreciating and Accommodating People With Communication and Behavior Challenges. Madison, WI: DRI press

[B6] Donnellan A. M. Leary M. R. Robledo J. P. (2006). I can't get started stress and the role of movement differences in people with autism, in Stress and Coping in Autism, eds Baron G. Groden J. Groden G. Lipsitt L. (Oxford: Oxford University Press), 200–245

[B8] Fabbri-Destro M. Cattaneo L. Boria S. Rizzolatti G. (2009). Planning actions in autism. Exp. Brain Res. 192, 521–525 10.1007/s00221-008-1578-3 18839160

[B9] Haswell C. C. Izawa J. Dowell L. R. Mostofsky S. H. Shadmehr R. (2009). Representation of internal models of action in the autistic brain. Nat. Neurosci. 12, 970–972 10.1038/nn.2356 19578379 PMC2740616

[B10] Iversen P. (2007). Strange Son Two Mothers, Two Sons, and the Quest to Unlock the Hidden World of Autism. New York, NY: Riverhead Books

[B11] Kanner L. (1943). Autistic disturbances of affective contact. Nerv. child 2, 217–250 4880460

[B11a] Klin A. Lin D. J. Gorrindo P. Ramsay G. Jones W. (2009). Two-year-olds with autism orient to non-social contingencies rather than biological motion. Nature 459, 257–264 10.1038/nature07868 19329996 PMC2758571

[B12] Lewis M. Kim S. J. (2009). The pathophysiology of restricted repetitive behavior. J. Neurodev. Disord. 1, 114–132 10.1007/s11689-009-9019-6 21547711 PMC3090677

[B13] Lord C. Risi S. Lambrecht L. Cook E. H. Jr. Leventhal B. L. DiLavore P. C. (2000). The autism diagnostic observation schedule-generic a standard measure of social and communication deficits associated with the spectrum of autism. J. Autism Dev. Disord. 30, 205–223 11055457

[B14] Lovaas O. I. (1987). Behavioral treatment and normal educational and intellectual functioning in young autistic children. J. Consult. Clin. Psychol. 55, 3–9 3571656 10.1037//0022-006x.55.1.3

[B15] Mizuno A. Villalobos M. E. Davies M. M. Dahl B. C. Muller R. A. (2006). Partially enhanced thalamocortical functional connectivity in autism. Brain Res. 1104, 160–174 10.1016/j.brainres.2006.05.064 16828063

[B16] Mostofsky S. H. Ewen J. B. (2011). Altered connectivity and action model formation in autism is autism. Neuroscientist 17, 437–448 10.1177/1073858410392381 21467306 PMC3974163

[B17] The American Occupational Therapy Foundation The American Occupational Therapy Association. (2011). Occupational Therapy Research Agenda. Available online at: www.aota.org/documentvault/research/45008.aspx [Accessed October 25, 2012].

[B18] Rizzolatti G. Fabbri-Destro M. (2010). Misrror neurons from discovery to autism. Exp. Brain Res. 200, 223–237 10.1007/s00221-009-2002-3 19760408

[B19] Schultz R. T. (2005). Developmental deficits in social perception in autism the role of the amygdala and fusiform face srea. Int. J. Dev. Neurosci. 23, 125–141 10.1016/j.ijdevneu.2004.12.012 15749240

[B20] Teitelbaum P. Teitelbaum O. Nye J. Fryman J. Maurer R. G. (1998). Movement analysis in infancy may be useful for early diagnosis of autism. Proc. Natl. Acad. Sci. U.S.A. 95, 13982–13987 10.1073/pnas.95.23.13982 9811912 PMC25000

[B21] Torres E. B. (2012). Atypical signatures of motor variability found in an individual with ASD. Neurocase 1–16 [Epub ahead of print]. 10.1080/13554794.2011.654224 22587379

[B22] Wallis C. (2006, May 7). Inside the autistic mind. TIME-NEW YORK-AMERICAN EDITION- 167, 42 17853568

[B23] Williams D. (1994). Nobody Nowhere. New York, NY: Harper Paperbacks

[B24] Zikopoulos B. Barbas H. (2007). Circuits for multisensory integration and attentional modulation through the prefrontal cortex and the thalamic reticular nucleus in primates. Rev. Neurosci. 18, 417–438 18330211 10.1515/revneuro.2007.18.6.417 PMC2855189

